# Diatom-Based Nanomedicine for Colorectal Cancer Treatment: New Approaches for Old Challenges

**DOI:** 10.3390/md21050266

**Published:** 2023-04-26

**Authors:** Chiara Tramontano, Luca De Stefano, Ilaria Rea

**Affiliations:** 1 Institute of Applied Science and Intelligent Systems (ISASI), National Research Council of Italy-Naples Unit, Via Pietro Castellino 111, 80131 Naples, Italy; 2 Department of Pharmacy, University of Naples Federico II, Via Domenico Montesano 49, 80131 Naples, Italy; luca.destefano@na.isasi.cnr.it

**Keywords:** chemotherapy, colorectal cancer (CRC), neoplasm metastasis, biosilica, diatoms, nanotechnology, nanomedicine

## Abstract

Colorectal cancer is among the most prevalent and lethal cancers globally. To address this emergency, countries have developed diffuse screening programs and innovative surgical techniques with a consequent decrease in mortality rates in non-metastatic patients. However, five years after diagnosis, metastatic CRC is still characterized by less than 20% survival. Most patients with metastatic CRC cannot be surgically treated. For them, the only option is treatment with conventional chemotherapies, which cause harmful side effects in normal tissues. In this context, nanomedicine can help traditional medicine overcome its limits. Diatomite nanoparticles (DNPs) are innovative nano-based drug delivery systems derived from the powder of diatom shells. Diatomite is a porous biosilica largely found in many areas of the world and approved by the Food and Drug Administration (FDA) for pharmaceutical and animal feed formulations. Diatomite nanoparticles with a size between 300 and 400 nm were shown to be biocompatible nanocarriers capable of delivering chemotherapeutic agents against specific targets while reducing off-target effects. This review discusses the treatment of colorectal cancer with conventional methods, highlighting the drawbacks of standard medicine and exploring innovative options based on the use of diatomite-based drug delivery systems. Three targeted treatments are considered: anti-angiogenetic drugs, antimetastatic drugs, and immune checkpoint inhibitors.

## 1. Introduction

**Figure 1:**
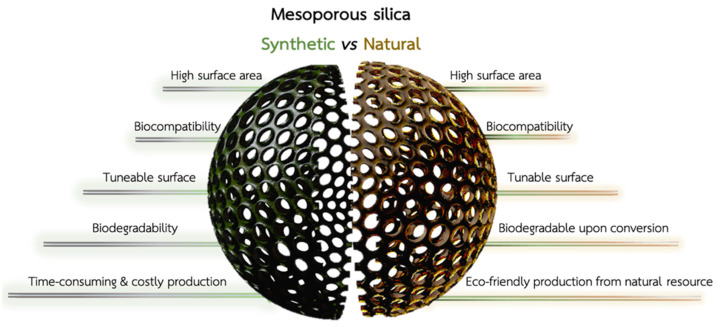
Comparison between synthetic mesoporous silica (green, left) and diatomite silica (brown, right). Synthetic and natural silica show common advantages, such as a high surface area, tunable surface chemistry, and biocompatibility for drug delivery. Diatom biosilica must be converted into biodegradable silicon for therapeutic applications, while synthetic mesoporous silica is naturally excreted from the body. However, the production of NPs from diatom biosilica is cheaper, eco-friendly, and, overall, more convenient than its synthetic counterpart.

The last 20 years have seen significant advances in treating metastatic colorectal cancer (CRC) due to the availability of new anticancer drugs targeting aberrant pathways [1]. The improved understanding of tumor biology has significantly broadened the therapeutic options, allowing for targeted therapy based on the underlying pathology [2]. The earliest phases of CRC start with abnormal cell replication in the mucosa and clusters of enlarged crypts with biochemical and biomolecular abnormalities [3]. Malignant cells pass through the muscular mucosae and infiltrate the submucosa, leading to macroscopic polypoid vegetating masses or flat infiltrating lesions. Most of these tumors are adenocarcinomas (96%) [4], while the rest include squamous carcinoma and solid carcinoma with minimal glandular differentiation or slight cellular pleomorphism [5]. Approximately 25% of CRC patients present with metastatic disease, and 18% of patients will develop metastases after treatment of the primary CRC [6]. The ideal CRC treatment aims to remove both tumor and metastases through endoscopic resection or surgical intervention. Unfortunately, only a few patients with metastatic CRC are likely to be cured and defeat cancer. Despite numerous screening tests available for adults between 45 and 70 years (e.g., stool tests, sigmoidoscopy, and colonoscopy) [7], nearly a quarter of CRC patients are diagnosed at an advanced stage with liver and non-isolated lung metastases [8]. Most patients cannot be cured through surgical resection, so their treatments are primarily based on palliative or systemic chemotherapy or radiotherapy [9]. Chemotherapy aims to suppress further tumor growth and metastases outbreak, prolong survival rate, and improve tumor-related symptoms. However, chemotherapy drugs have shortcomings in clinical application, such as limitations due to poor water solubility, serious adverse effects [5] caused by non-specific distribution, infections, and permanent damage to vital organs. Advanced technological approaches based on gene therapy [10] and immunotherapy [11] are changing the pattern of tumor treatment. Nevertheless, low cell uptake rate and poor tumor permeability seriously affect their translation into clinics. Nanotherapeutics with improved retention time and reduced toxicity show great promise for treating CRC, with decisive results in the future [12]. The growing interest in applying nanoparticles (NPs) to cancer is related to their ability to deliver a drug to the site of action, protect it from degradation, and overcome both pharmacokinetic and collateral effects [13]. So far, fifty nanomedicine formulations have reached the market. Three new products (Onpattro^®^, Hensify^®,^ and Vyxeos^®^) [14] have recently been approved, showing that nanomedicine overcomes critical barriers in conventional medicine and improves patients’ lives. In this scenario, mesoporous silica nanoparticles capable of retaining drugs inside their pores have gained increasing attention for the controlled release of anticancer drugs [15]. The possibility to modify mesoporous silica NPs with both polymers and targeting moieties resulted in the fabrication of nanotherapeutics with a site-specific drug release triggered by unique stimuli in the cancer microenvironment (stimuli-responsive) [16]. Biogenic nanostructured porous silica can be obtained from the powder of diatom shells (diatomite), which can be reduced into mesoporous silica NPs with a low-cost and eco-friendly approach [17] (Figure 1). Diatomite NPs (DNPs) can be produced through the ultrasonication of diatom shells (frustules) in ethanol, which allows for the reduction of the micrometer frustules into NPs with a size distribution between 300 and 400 nm [18]. Then, the DNPs undergo two sequential purification processes with Piranha (4 H_2_SO_4_:1 H_2_O_2_) and HCl 5M solutions to remove the organic and inorganic pollutants, respectively [19,20]. The as-obtained NP suspension can then be characterized morphologically and functionalized as needed. Diatom biosilica is rich in reactive silanol groups, which can be further conjugated to biological moieties (e.g., proteins and antibodies) for targeted NP uptake and drug release [18,21]. Tramontano et al. described different approaches to functionalize and load chemotherapeutic drugs into DNPs for sustained drug release in cancer cells [18]. Targeting specific antigens on the cell surface is critical to strengthening the therapeutic efficacy of the delivered drug, enhancing therapeutic efficacy, and reducing side effects (Figure 1). Moreover, being obtained from a natural fossil source, the Food and Drug Administration (FDA) has already approved diatom biosilica for pharmaceutical and animal feed formulations (GRAS 21CFR 573.340). Unlike synthetic silica nano/microparticles, which are expensive and non-ecological (Figure 1), diatom biosilica can be produced in large amounts without costly materials and energy [22]. Diatomaceous biosilica can be harvested from caves and reduced at the nanoscale using different techniques, which vary significantly from case to case [23]. Therefore, it is necessary to investigate the scale-up feasibility of nanocarriers with specific biomedical applications. However, the approval of diatom biosilica for therapeutic purposes is yet to be issued due to its non-biodegradability. To overcome this challenge, the conversion of DNPs (silica) into silicon replicas through magnesio-thermal reduction was proposed and successfully reported in the literature [24] (Figure 1). Due to its intriguing properties, diatom biosilica is a promising material to produce low-cost and biocompatible nanocarriers carrying chemotherapeutic drugs to cancer cells with high specificity. 

In this short review, we briefly explore the traditional approach (chemotherapy) and primary targets (angiogenesis and immunological checkpoints) involved in treating metastatic CRC, emphasizing the advantages and drawbacks of conventional medicine. We then give an overview of the applications of diatom-derived biosilica as nanocarriers reported in the last decade to underline up-and-coming novel perspectives for the targeted treatment of metastatic CRC. The studies in this review paper prove that diatom-based nanocarriers with enhanced internalization and controlled release properties can be made by combining traditional drugs with stimuli-responsive polymers and targeting moieties (e.g., antibodies). Nevertheless, despite the numerous advantages of porous diatom biosilica, the clinical development of diatomite-based nanocarriers for CRC treatment still needs to be improved, mainly due to the need for pre-clinical studies. Therefore, we stress the necessity to investigate the potentiality of this material in other intricate systems to decode its potential in the treatment of cancer and, more specifically, CRC.

## 2. Nanomedicine: New Ways to Face Old Challenges

To manipulate matter on the atomic/molecular scale on demand was an old dream of natural science, and it came true in 1960 with the production of the first nanomaterials [25]. Ever since, nanotechnology has been introduced into our daily routine, opening up alternative approaches in optics, electronics, chemistry, pharmaceutics, engineering, and medicine [26]. The application of nanotechnology to medicine is defined as nanomedicine, which consists of using nanomaterials to prevent, diagnose, follow up, and treat disease. The European Medicines Agency (EMA) defines nanomedicines as products with at least one component on the nanometer (10^–9^ m) scale with specific properties determined by medical needs and associated with the clinical advantages of nano-engineering [27]. Nanomaterials differ from their bulk counterpart due to the high volume-to-surface area ratio and generation of possible quantum effects, enabling various applications in medicine [28]. 

Nanotherapeutics consists of drug delivery systems capable of increasing drug availability at the tumor site and improving therapeutic efficacy. Theranostics has emerged as a promising approach offering diagnosis and therapy simultaneously within the same system [29]. The success of nanotechnologies in drug delivery can be explained by the recognized advantages in clinics, such as increased drug distribution, improved pharmacokinetics, overcoming of critical barriers, such as kidney filtration and reticuloendothelial system clearance [30]. NPs possess extraordinary advantages over traditional medicine as they enable the delivery of insoluble drugs previously undevelopable due to water instability and protect drug cargo from enzymatic degradation or pH inactivation. Furthermore, the controlled drug release increases bioavailability, allowing targeted drug delivery while reducing side effects. Identifying immunologically inert materials eliciting the desired functions has resulted in the fabrication of a vast collection of NPs, usually classified by their composition as organic, inorganic, and hybrid [31]. Each class of NPs includes different subclasses with unique size, design, surface area, and chemistry [32]. Researchers have also developed hybrid systems from organic and inorganic NPs [23]. Lipid-based NPs can load hydrophobic and hydrophilic drugs, representing the most common class of FDA-approved nanomedicine products [33]. Liposomes carrying medicinal drugs such as daunorubicin (DaunoXome^®^) and Doxorubicin (Doxil^®^) have been approved for treating CRC. Similarly, Thermodox^®^ (thermo-sensitive liposome-DOX) is used for CRC liver metastasis treatment in combination with radiofrequency ablation [34]. In this treatment, Doxil^®^ releases DOX in response to mild hyperthermic signals with a release efficiency twenty-five-fold higher than conventional administration. 

The exploration of more robust nanomaterials for drug delivery purposes turned the spotlight on inorganic nanomaterials, such as silica [35], silicon [36], gold [37], and iron NPs [38]. Among these, silica (SiO_2_) NPs—the oxidized form of silicon—have found wide application in drug delivery due to their porous structure, allowing for the entrapment of a higher amount of drug cargo than organic NPs [16]. Moreover, due to their robustness, inorganic NPs are stable in body fluids for longer periods, serving purposes otherwise unattainable by other types of nanomaterials. 

## 3. Standardized and Groundbreaking Approaches to Fight against Colorectal Cancer

### 3.1. Commonly Used Chemotherapeutics in Metastatic Colorectal Cancer

Since its introduction into clinical trials in 1950, 5-fluorouracil (5-FU) has represented the cornerstone of palliative and adjuvant treatments of CRC. 5-FU is an antimetabolite drug that inhibits the thymidylate synthase (TS), interfering with normal DNA and RNA functions and inducing cytotoxicity [39]. 5-FU is administered intravenously (IV) as a rapid continuous bolus infusion with leucovorin (LV) or as the oral prodrug capecitabine [40], which is converted into 5-FU by the thymidine phosphorylase (TP) in tumor sites. Due to an increased expression of TP in the cancer microenvironment, the concentration of 5-FU in tumors was found to be 3.2-fold higher than in normal tissues [41]. Randomized phase-III studies [42] showed that although a lower incidence of side effects is observed with capecitabine, the efficacy of the prodrug is comparable with 5-FU/LV. The results reported in recent decades show that chemotherapy has pushed the survival time of patients to 20 months after diagnosis, resulting in the most effective treatment for metastatic CRC. However, cytotoxic agents are often associated with systemic toxicity, unsatisfying response rate, and multi-drug resistance. 

**Figure 2:**
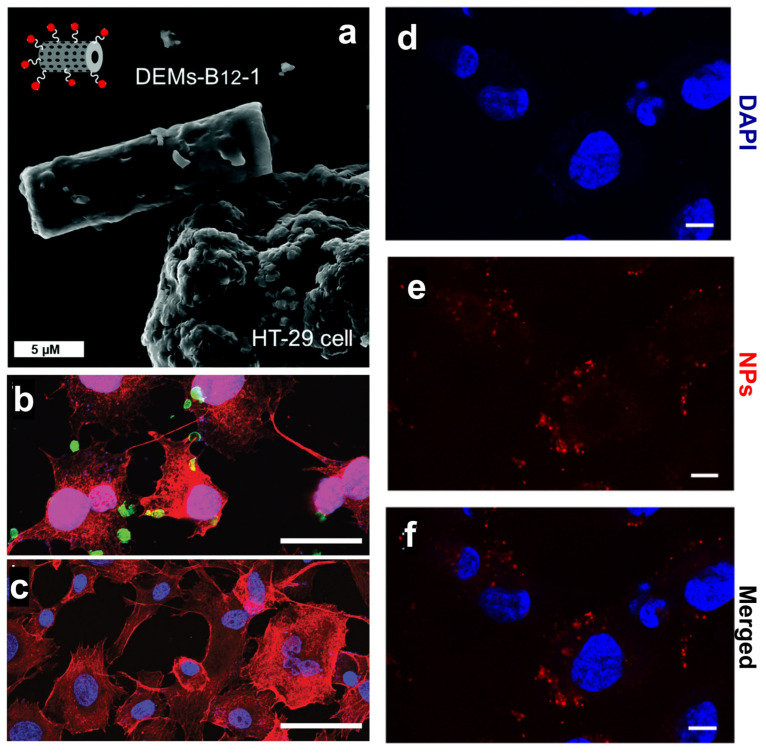
(**a**) SEM investigation of DEM-B_12_ interaction with HT29 cells mediated by the transcobalamin receptor. (**b**,**c**) Specific uptake of genetically engineered diatoms modified with the anti-CD20 antibody in cancer cells (**b**) and control cells (**c**). Scale bars are 50 µm (**b**,**c**). (**d**–**f**) Uptake of PSiNPs-DOX obtained from diatom biosilica in cancer cells. Cell nuclei were stained with DAPI (**d**), while the PSiNPs-DOX exhibited a natural red fluorescence (**e**). Merged channels are shown in (**f**). Scale bars are 25 µm (**d**–**f**). Adapted with permission from [43] (**a**), [47] (**b**,**c**), and [24] (**d**–**f**).

To overcome the pharmacokinetic issues of 5-FU, Delasoie et al. proposed 10 µm diatom microalgae (DEM) loaded with either 5-FU or a novel lipidic ruthenium (II) complex and further modified with vitamin B_12_ (DEM-B_12_) targeting HT29-MTX and MCF-7 cancer cells [43]. DEMs were not expected to penetrate cancer cells but rather interact with them via the transcobalamin receptor expressed on the cell membrane (Figure 2a) and promote a site-specific drug release. The authors demonstrated the ability of DEM-B_12_ to release both the water-soluble 5-FU and lipidic ruthenium complex in cancer cells thanks to an efficient interaction with the transcobalamin receptor [43] (Table 1). The same group showed that a two-fold increase in cytotoxicity toward colorectal HCT-116 cells could be achieved by chemo-photodynamic therapy with DEM-B_12_ carrying rhenium (I) tricarbonyl complexes and photoactivable molecules releasing CO_2_ [44] (Table 1). Nanomedicine aims to decrease the administered dose by concentrating the drug in the targeted site, thus lowering the side effects in normal cells and increasing patients’ compliance [45]. The synergistic effect of the delivered drug and released CO_2_ provided the formulation developed by Delasoie et al. [44] with increased efficacy against the CRC cell line, thus reducing the necessary therapeutic dose [46]. 

The ability of diatom-based nanocarriers to selectively target cancer cells was also explored by Delalat et al. with genetically engineered diatoms carrying liposomes loaded with camptothecin (CPT) [47]. The authors encapsulated the drug into positively charged liposomes with a loading capacity of up to 85% to solve the challenging task of loading the hydrophobic CPT. Then, the drug-loaded liposomes were absorbed on the surface of diatoms expressing the anti-CD20 antibody that targets a highly expressed cell surface marker of B cells. The active targeting strategy resulted in a selective killing of neuroblastoma cells (Figure 2b), while minimal effects were observed in the healthy cell line **(**Figure 2c). Biodistribution experiments showed that both functionalized and non-functionalized biosilica was observed in the liver and kidney because of particle uptake by macrophages in the reticuloendothelial systems [47]. Earlier studies have shown that the accumulation of silica NPs in crucial organs, such as the kidneys, liver, spleen, and lungs, increases the activity of liver enzymes (AST and ALT) in male rats [48], causing hepatic damage and affecting membrane permeability with consequent enzyme leakage from hepatocytes (acute toxicity). The active targeting approach proposed by Delalat et al. induced a substantial reduction in tumor growth in vivo with a single dose of nanocarriers, whereas CPT alone had a negligible effect at the same concentration as the NPs [47]. In most cases, single-agent therapy based on CPT administration is insufficient at controlling tumor growth, requiring the combination of chemotherapeutic drugs with small molecules inhibiting signaling pathways [49]. Although oral doublet therapy is worthy of continued investigation for the treatment of metastatic CRC, treatment resistance may develop, as for the combination of sorafenib with CPT and doxorubicin with tamoxifen [50,51]. To overcome these issues, DNPs were proposed as nanocarriers of sorafenib to increase its therapeutic efficacy and avoid the systemic administration of two drugs. Terracciano et al. proposed DNPs 400 nm in size loaded with sorafenib and modified with a cell-penetrating peptide (CPP) for enhanced cell penetration and NP uptake [52] (Table 1). Although the authors tested the efficacy of the NPs in MCF-7 and MDA-MB-231 breast cancer rather than CRC cells, they showed a suitable strategy to increase the uptake of the NPs and promote sorafenib accumulation and cytotoxic effect in CRC exhibiting chemoresistance.

To reduce the hematological toxicity caused by doxorubicin (DOX), Maher et al. converted diatom silica into silicon NPs in which DOX was encapsulated (PSiNPs-DOX) (Table 1). The authors compared the efficacy of the delivered drug to free DOX in the cancer cell line (RAW 246.7) [24]. Thanks to the consistent interaction of the fluorescent (red) NPs with the cells (Figure 2d–f), the authors showed that DOX released from PSiNPs-DOX (50 µg/mL) exhibited cytotoxicity that was two-fold stronger than the free drug, allowing a decrease in the administered DOX dose and toxicity as well. Diatoms are rich in biosilica and primary and secondary metabolites (fatty acids, esters, acyl lipids, sterols, proteins, and flavonoids) with anti-cancer activity [53]. The monoacylglycerides (MAG) isolated from the diatom *Skeletonema marinoi* exhibit potent anticancer effects on HCT-116 colon cancer cells through the induction of apoptosis with an IC_50_ of 5 µg/mL after 24 h treatment and without affecting normal cells [54]. The absorption ability of MAG in cancer cells confirms their ability to prevent tumor genesis and highlights the opportunity of combining the anticancer efficacy of diatom metabolites with the potentiality of the delivered drug to halt cancer growth, progression, and metastases.

**Table 1:** Diatom-based drug delivery systems for the treatment of either metastatic or non-metastatic cancer.

Authors	Delivered Chemotherapeutic Drug	Strategy	Drug Loading (*wt*)	In Vitro Cancer Model	In Vivo Biosilica Biodistribution
Zobi et al. [43]	5- fluorouracil (5-FU)	B_12_-modified microparticles (DEMs)	~7.3%	HT-29	N/A
Delalat et al. [47]	Camptothecin (CPT)	Drug-loaded liposomes on anti-CD20-modified diatoms	~85%	SHSY5Y, BSR	Lungs, liver
Terracciano et al. [52]	Sorafenib	CPP-modified DNPs	~22%	MCF-7, MDA-MB-231	N/A
Maher et al. [24]	Doxorubicin (DOX)	Silicon NPs obtained from diatoms	~31%	RAW 246.7	N/A
Rea et al. [19]	Small interfering RNA (SiRNA)	Poly-Arg-modified DNPs	N/A	H1355	N/A
Zhang et al. [55]	Mesalamine	Diatom MPs	~11.5%	Caco-2, HT-29	N/A
Sasirekha et al. [56]	DOX	Chitosan-modified frustules	~27%	Caco-2, HT-29, HCT-116	N/A
Todd et al. [38]	Dyes	IONP-embedded diatoms	N/A	4-T1	Lungs (narrow capillaries)
Managò et al. [57]	Galunisertib (LY)	Gelatin-coated gold-decorated DNPs	~2%	SW620, LS17-4T	N/A
Tramontano et al. [35]	Galunisertib (LY)	Antibody-modified DNPs-LY encapsulated in HPMC	~5%	Caco-2, HT29-MTX, SW620	N/A
Ibrahim et al. [58]	Ibuprofen	Chitosan-diatomite nanocomposites	~56%	N/A	N/A
Terracciano et al. [59]	Anti-PD-L1 peptide nucleic acid	Redox-responsive DNPs	~31%	A549	N/A
Martucci et al. [60]	Anti-Bcl-2 small interfering RNA	Id-peptide-modified DNPs loaded with siRNA	N/A	A20	N/A

### 3.2. Novel Approaches and Molecular Markers for Targeted Treatments of Metastatic Colorectal Cancer

The increasing knowledge of the pathways involved in CRC growth, progression, and metastases has led to targeted therapies with improved therapeutic outcomes and reduced side effects. Targeted approaches involve the use of a drug precisely targeted to an “invader” (e.g., proteins, nucleic acids) whose function (or dysfunction) promotes cancer cell proliferation and migration [61]. Turning off or regulating a dominant signal in CRC with the drug may increase tumor cell killing and decrease off-target effects, such as epithelial cell damage [62]. For example, almost 40% of cases of metastatic CRC are caused by mutations in members of the RAS superfamily [63], resulting in increased proliferation, angiogenesis, and alterations of cell metabolism. In other cases (approximately 20%), CRC is caused by mutations in the epidermal growth factor (EGF) gene [64], which plays a crucial role in tumorigenesis or hyperactivation of the transforming growth factor (TGF) receptor [65]. Due to the dynamicity of the disease causes, numerous efforts have been made to identify the main pathways involved in CRC genesis and progression. This review will cover three main targeted approaches for metastatic CRC, including antiangiogenetic and antimetastatic drugs and immune checkpoint inhibitors.

#### 3.2.1. Targeting Angiogenesis to Inhibit Cancer Proliferation and Migration

The populations of growing cancer cells, capillary endothelial cells, and immune cells (macrophages, lymphocytes) constitute a highly integrated network depending upon each other in solid tumors. To grow rapidly, cancer cells require oxygen, nutrients, and shuttles toward distant organs, provided by the crazy-quilt growth of blood vessels in a process known as angiogenesis [66]. Hypoxia [67] and a lack of nutrients [68], which are the significant consequences of misbalanced proliferation, bolster the expression of cytokines and the transcription of the vascular endothelial growth factor (VEGF) and platelet-derived growth factor (PDGF) [69]. In this process, the matrix metalloproteinases (MMPs) disrupt the extracellular matrix (ECM), enabling cells to penetrate the underlying stroma and migrate toward distant sites. Due to the dominant contribution of angiogenesis in the progression of metastatic CRC, targeting the pro-angiogenic factors is an effective strategy for managing CRC [70].

**Figure 3:**
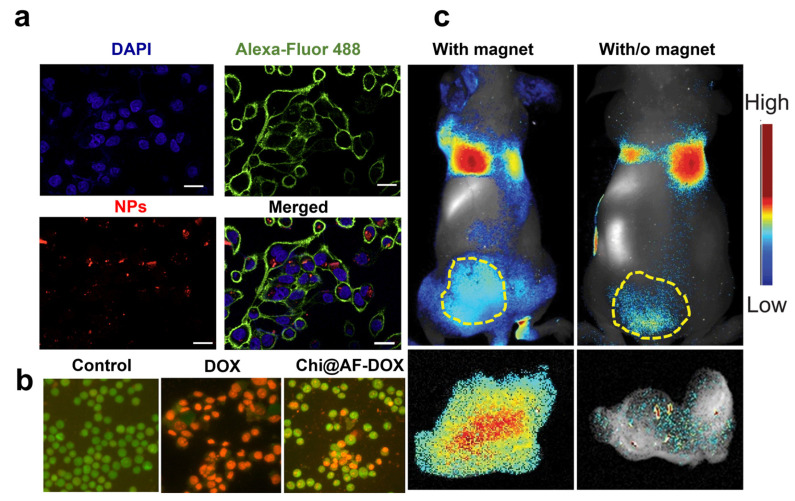
(**a**) Confocal microscopy on cells treated with siRNA*-modified diatomite nanovectors labeled with Dy547 (NPs). Cell nuclei and membranes were stained with Hoechst 33342 and WGA-Alexa Fluor 488, respectively. The scale bar is 20 μm. (**b**) A549 cells incubated for 24 h with PBS (control), bare frustules (AF), and Chi@AF-DOX at a concentration of 1 μg/mL (**c**) and 37 °C for 24 h. AOEB was used for staining the apoptotic bodies of cells, while green fluorescence represents viable cells and yellowish-orange cells represent apoptotic cells. (**c**) In vivo imaging results of IONP-embedded diatom accumulation in mice with (left) and without (right) the application of an external magnetic field. During the process, a higher signal (6.4 times stronger) of NP accumulation was observed in the tumors attached to a magnet. Reproduced and adapted with permission from [19] (**a**), [56] (**b**), and [38] (**c**).

The era of anti-angiogenic treatments started with the approval of bevacizumab (Avastin^®^), a humanized monoclonal antibody targeting VEGF-A and depleting tumor vasculature [71]. The FDA approved other proteins targeting angiogenesis as a second-line strategy for treating metastatic CRC in patients who are refractory to chemotherapy. These include the soluble recombinant fusion protein Ziv-aflibercept (Zaltrap^®^) [72], regorafenib (Stivarga^®^) [73], and the fully humanized immunoglobulin IgG1 ramucirumab (Cyramza^®^) targeting the VEGF receptor 2 extracellular domain [74]. Suppressing angiogenesis in CRC by inhibiting the VEGF/VEGFR signaling pathway offers a fundamental approach to cancer therapy. Only a few studies support antiangiogenic drugs in monotherapy, as they are most often used with other regimens with increased risks of toxicity and side effects. Toxicities of antiangiogenic proteins include hypertension, proteinuria, thrombosis, gastrointestinal dysfunction, and perforation, highlighting the need for alternative routes to block the angiogenesis pathway [75]. Small interfering ribonucleic acid (RNA) has recently reinforced the therapeutic strategies against cancer angiogenesis, allowing for the sequence-specific post-transcriptional downregulation of genes involved in the angiogenesis cascade [76]. Small interfering RNA targeting a novel tropomyosin isoform TC-22 associated with metastatic CRC reduced protein levels and angiogenesis by 45 to 50% [77]. However, although siRNAs can target overexpressed proteins with higher efficiency than antibodies, they may modify cell functionalities and cause non-specific toxicity [78]. Moreover, unmodified siRNAs have unsatisfactory stability in body fluids with fast nuclease degradation and a poor pharmacokinetic profile, limiting their access to tumor tissues. To overcome these issues, Rea et al. proposed poly-arginine-modified DNPs as innovative nanocarriers for siRNA transport into human H1355 epidermoid carcinoma cells with efficient gene silencing of the target protein [19]. First, the authors showed the ability of DNPs to protect the siRNA complex from degradation by human pancreatic ribonuclease, improving SiRNA stability and lifetime. Then, the cells were incubated with free siRNA and 300 µg/mL of the nanocarrier (DNPs-SiRNA), delivering the same drug concentration. Effective uptake of the engineered nanovector in the cell cytoplasm was shown through confocal microscopy (Figure 3a) alongside a promising downregulation of the target, overcoming the issue of traditional siRNA-based therapies [19]. The orally administered drug mesalamine downregulates TC-22 and angiogenesis, potentially inhibiting angiogenesis and preventing cancer [77]. Zhang et al. developed diatom microparticles (MPs) 4–6 µm in diameter for the controlled delivery of mesalamine in the colon, preventing drug metabolization in the liver and increasing biocompatibility [55]. Mesalamine was loaded into the MPs with an efficiency of 11.5% *wt* and released in Caco-2/HT29 co-cultures with enhanced drug permeation from 16 to 33%. The developed microcarrier showed no toxicity in CRC cells up to concentrations as high as 1000 mg/mL, suggesting that DMPs are a promising material for the oral delivery of drugs inhibiting angiogenesis in CRC [55]. Recent studies supported the proposal that sulfated chitosan could block the VEGF/VEGFR pathway with higher inhibitory effects on tumor angiogenesis and lower toxicity than heparin [79]. Therefore, chitosan-modified diatom-based approaches were also proposed to inhibit cancer growth and metastases dissemination in CRC. Sasirekha et al. obtained nanostructured biosilica from *A. subtropica* diatoms (AF). They loaded DOX into the porous structure of chitosan-modified frustules (Chi@AF) to achieve the inhibition of both tumor growth with DOX and angiogenesis with chitosan (Chi) [56]. The synergistic targeting of cancer proliferation and angiogenesis resulted in a more potent cytotoxic effect on A549 cells through apoptosis within 24 h of incubation with the drug-loaded diatoms. Cells in late apoptosis treated with Chi@AF-DOX showed orange-yellowish nuclei and condensed or fragmented chromatin. In contrast, control cells (untreated) showed green fluorescence with uniform and intact cell membranes (Figure 3b). No apoptotic effect was observed in the presence of the diatom frustules AF, confirming silica biocompatibility. However, apoptosis induced by the equal concentration of free DOX (Figure 3b) was higher than the Chi@AF-DOX, suggesting that further formulation improvements are necessary in order to enhance the inhibition of cancer angiogenesis [56]. 

A novel method to increase the accumulation of diatom biosilica in metastatic tumors was investigated by Todd et al. with iron oxide NP (IONP)-embedded diatoms for the magnetic delivery of small molecules in vivo [38]. Diatom frustules (10 µm) were embedded with hundreds of magnetic NPs granting biosilica a superior magnetic response after intravenous injection into the 4T1 tumor xenograft model (1.65 mg·kg^−1^). The authors showed that the accumulation of IONP diatoms in the tumor tissue could be enhanced by applying an external magnetic field (e.g., magnetic bar) to the skin, yielding over 6.4 times more particles than without the magnetic bar (Figure 3c). The improved tumor retention, low toxicity, and degradability of both IONPs and diatoms underline the great potentialities of diatom biosilica for metastatic CRC with minimal invasiveness in normal tissues. 

#### 3.2.2. Targeting Metastases Outbreak and Dissemination in Metastatic Colorectal Cancer

**Figure 4:**
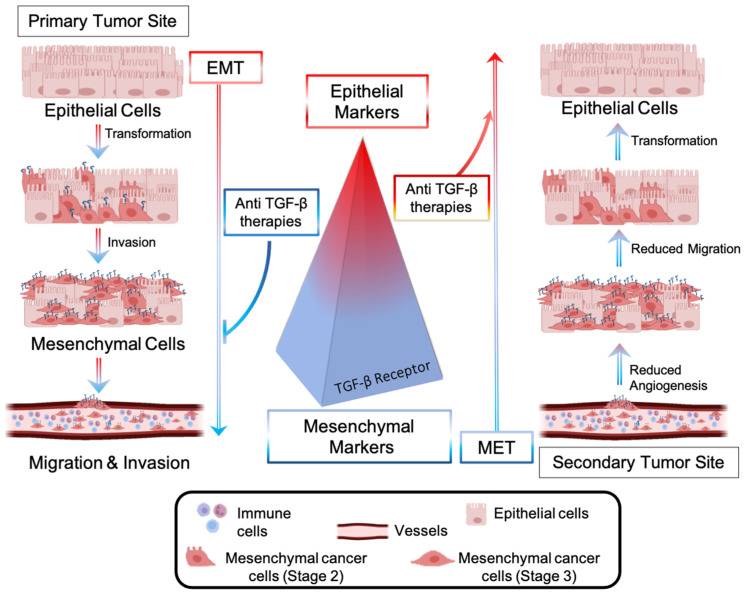
Epithelial-to-mesenchymal transition (EMT) is driven by CRC cells’ transforming growth factor (TGF-β). Upregulation of TGF-β signaling promotes overexpression of mesenchymal markers, which favor EMT, migration, and invasion of CRC cells into a secondary tumor site. Anti-metastatic approaches include the blockade of the TGF-β-mediated pathway, inhibition of EMT, and promotion of a molecular and morphological cell transformation known as the mesenchymal-to-epithelial (MET) transition.

Metastases represent a significant barrier to the favorable outcome of cancer treatments, and, therefore, tremendous efforts have been made to develop drugs interfering with the metastatic pathway [80]. The dissemination of CRC cells from a primary to a secondary tumor site involves the local invasion of circulating tumor cells (CTCs) into adjacent tissues, extravasation, and colonization of distant organs [81]. Cancer cells are intimately connected to any component of the tumor microenvironment and release growth factors, such as VEGF, to create a plexus of blood vessels within the tumor. The epithelial to mesenchymal transition (EMT)—an evolutionarily conserved development program—contributes to carcinogenesis and metastases outbreaks by enhancing cell mobility, extravasation, and dissemination [82]. During the EMT, cancer cells undergo a morphological and molecular transformation into spindle-shaped cells (also referred to as EMT-like or mesenchymal phenotype) and express mesenchymal markers that favor their dissemination and invasion (Figure 4) [82]. Among the receptors involved in the regulation of the EMT process, the transforming growth factor-β (TGF-β) receptor (R) promotes the upregulation of metastatic genes (*Snail*, *Twist*, *Vimentin*) and downregulation of epithelial shape (*E-Chaderin*) [83]. Anti-TGF-β therapies consist of blocking the metastatic pathway driven by TGF-β-R and inducing the mesenchymal-to-epithelial (MET) transition, reducing cell invasiveness and aggressiveness (Figure 4). Several non-cytotoxic anti-TGF-β inhibitors have recently been brought into clinics for patients with invasive CRC and with metastases to crucial organs. They include biologics (fresolimumab [84], bintrafusp alpha [85]) and oral small molecules, such as galunisertib [86]. Oral anticancer drugs with a small molecular structure display fast liver metabolization, which decreases drug bioavailability at the target site, thus requiring multiple administrations [87]. 

**Figure 5:**
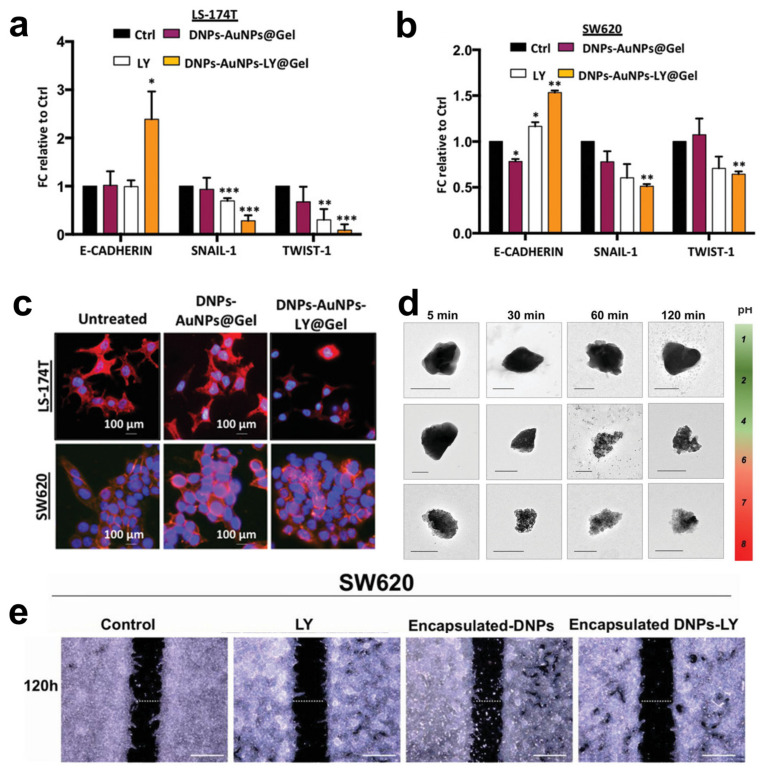
(**a**,**b**) Quantitative polymerase chain reaction (qPCR) analysis of EMT genes in LS-174T and SW620 cells incubated with 2.5 × 10^−6^ M LY, 50 μg mL^−1^ DNPs-AuNPs@Gel (empty), and DNPs-AuNPs-LY@Gel (releasing 2.5 × 10^−6^ M LY). Data were normalized to GAPDH expression and presented as fold change (FC) in gene expression relative to control. * *p* < 0.05, ** *p* < 0.005, *** *p* < 0.0005. (**c**) Representative images of DNPs-AuNPs-LY@Gel-mediated MET in LS-174T and SW620 cells. The scale bars are 100 μm. (**d**) Transmission electron microscopy (TEM) images of the encapsulated DNP dissolution at different pHs mimicking the GI. The scale bars are 300 nm. (**e**) Migration assay of SW620 cells incubated with 0.5% FBS DMEM (control), 2.5 µM LY, 26 µg mL^−1^ encapsulated DNPs (empty), and drug-loaded encapsulated DNPs in 0.5% FBS DMEM for 120 h. The scale bars are 500 µm. Adapted with permission from [57] (**a**–**c**) and [35] (**d**).

Managò et al. encapsulated galunisertib (LY) in plasmonic-assisted gelatin-coated DNPs (DNPs-AuNPs-LY@Gel) to overcome drug metabolization and promote a pH-triggered drug release with efficient NP tracking [57]. Drug release in the cells was triggered by gelatin degradation by the matrix metalloproteinases (MMPs) overexpressed in CRC. This suggests a practical approach to concentrating the drug in cancer cells overexpressing the stimulus. Furthermore, the high sensitivity and reliability of the plasmonic DNPs-AuNPs allowed the authors to trace drug release in living cancer cells for 48 h with an unprecedented sub-femtogram resolution. The increased galunisertib concentration in LS17-4T and SW620 CRC cells turned off the metastatic signaling (Snail, Twist) with higher efficiency than the free drug, promoting metastatic reversion by the induction of epithelial genes (Figure 5a,b). The metastatic reversion in LS17-4T and SW620 was confirmed by a well-evident cell phenotype change from spindle-shaped (aggressive) to rounder cells with reduced capability of colonizing a secondary tumor site (Figure 5c). Hybrid DNPs decorated with PEG-stabilized AuNPs (PEG-DNPs@AuNPs) and characterized by an average size of 400 nm were proposed by Terracciano et al. as effective contrast agents in imaging (e.g., photoacoustic, X-ray) [88]. The hybrid PEG-DNPs-AuNPs were internalized in HeLa cancer cells and localized in the perinuclear region. Cytotoxicity tests performed by incubating HeLa cells with PEG-DNPs@AuNPs up to 72 h confirmed a cell viability higher than 80%, ruling out any cytotoxic effect of the AuNPs on the cells.

Combining inorganic nanomaterials, such as DNPs, with organic polymers is a valuable method to develop hybrid multifunctional systems with degradation and drug release profiles controlled by the organic composition [89]. Tramontano et al. showed that polymer coating could prevent both the drug loading and release capacity of DNPs [90]. The authors dispersed drug-loaded DNPs into gelatin solution with concentrations between 0.1 and 1.5% *w/v*, reporting an enhanced drug loading capacity (from 2 to 6%) provided by a thicker gelatin shell surrounding the NPs. The release of the drug was controlled by the enzymatic degradation of the polymer shell, achieving a slower galunisertib release with thicker polymer shells [90]. The same group reported a microfluidic approach to encapsulate antibody-modified galunisertib-loaded DNPs into the FDA-approved hydroxypropyl methylcellulose (HPMC) polymer for oral treatment of metastatic CRC [35]. Oral administration of DNPs is preferred to the intravenous route because it enables galunisertib to be concentrated in the colon, avoiding particle clogging in blood vessels and drug shuttling and metabolization in the liver [91]. The efficient encapsulation of antibody-modified DNPs (encapsulated DNPs-LY) in the gastro-resistant polymer protected against gelatin degradation in stomach conditions (pH 1.6 and pepsin) and favored drug release in the intestine and colon environment (pH > 5.5 and trypsin) (Figure 5d) [35]. After dissolution in the intestine, the antibody-modified DNPs penetrated the metastatic SW620 cells overexpressing L1-CAM through antigen–antibody interactions, yielding increased NP accumulation and drug release compared to non-metastatic cells. As a result of the simultaneous L1-CAM and TGF-β targeting, the developed encapsulated DNPs-LY carrying galunisertib 2.5 µM inhibited the migration of CRC cells with higher efficiency than free drug at an equal concentration (Figure 5e) [35]. Ibrahim et al. proposed chitosan-modified diatomite nanocomposites for ibuprofen delivery at gastric and intestinal pH overcoming drug degradation in the mouth and metabolization in the liver [58]. The unique porous feature of diatoms inspired the development of diatom-like silica-coated human albumin NPs for the delivery of ruthenium polypyridyl (Ru 3) molecules to halt liver metastases through photodynamic therapy (PDT) [92]. The diatom-like structure protected Ru3 molecules from light inactivation and promoted the generation of ROS species from the NPs in HepG cancer cells. Moreover, the delivered Ru3 complexes showed excellent photodynamic properties in both HepG and A549 cells, substantially reducing cancer cell viability (Table 1).

Overall, diatom-based nano/microcarriers were extensively investigated for the release of low water-soluble compounds (anti-cancer, antibiotics, contrast agents) through oral administration due to the opportunity to avoid drug precipitation and crystallization into the porous silica structure. However, only a few studies investigating diatom-based nanomedicine for inhibiting CRC metastases are reported due to the inefficiency of the delivered anti-metastatic drugs to inhibit both cell growth and metastases. The co-delivery of cytotoxic and anti-metastatic drugs from diatoms or diatomite nanocarriers can help overcome this constraint, fostering a new approach to inhibiting cancer proliferation and dissemination simultaneously.

#### 3.2.3. Targeting Immunological Checkpoints to Activate Immune System

**Figure 6:**
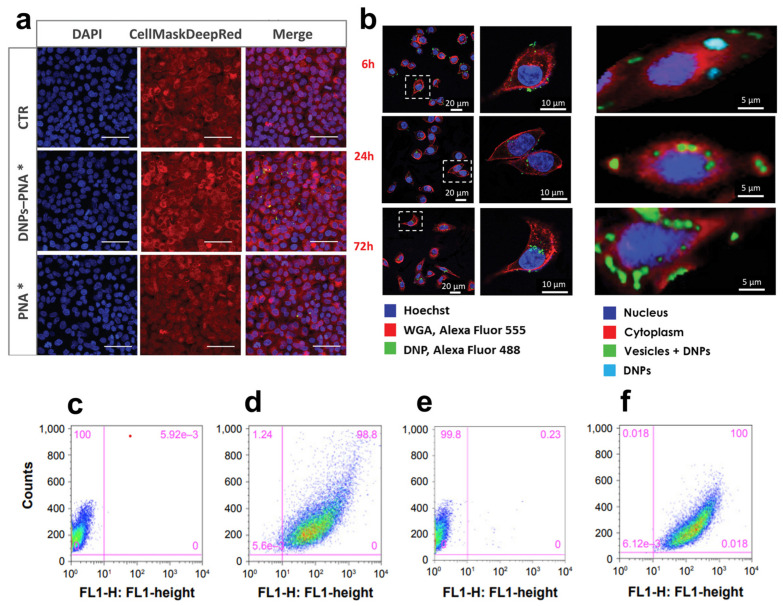
(**a**) Confocal microscope images of A549 cells incubated with DNP–PNA* or PNA* for 24 h. The cell membrane was stained with CellMask Deep Red, while the cell nuclei were stained with DAPI. The images were acquired using a 63× objective. Scale bar is 50 µm. (**b**) DNP uptake kinetics in cancer cells though confocal (left) and Raman imaging (right) at different incubation times (6, 24, 72 h). (**c**–**f**) Fluorescence-assisted cell sorting (FACWS) analysis of non-permeabilized A20 cells treated with PBS (control, **c**,**e**) or DNPs-siRNA (**d**,**f**). Adapted with permission from [59] (**a**), [104] (**b**), and [60] (**c**–**f**).

The response of immune checkpoints to tumors plays a significant role in the early stage of carcinogenesis, recognizing and killing cancer cells with abnormal features and uncontrolled growth rates [93]. The elimination of cancer cells occurs via the presentation of tumor antigens to the T-cell receptor (TCR), which is further activated by the binding of co-stimulators, resulting in efficient tumor eradication by T-cells. This process, known as cancer immune surveillance, relies on activating many immunological checkpoints, including the programmed cell death-1 receptor (PD-1) [94]. PD-1 is an inhibitor of adaptive and innate immune responses and is expressed in many immune cells, including activated T-cells [95]. PD-L1, in turn, is overexpressed by tumor cells as an “adaptive immune mechanism” to escape anti-tumor eradication [96]. The binding of PD-L1 on cancer cells to PD-1 on T-cells acts as a pro-tumorigenic factor inhibiting their activation, inducing EMT and the stem cell-like phenotype, and promoting cancer progression [97]. The inhibition of immune checkpoints through monoclonal antibodies, such as nivolumab (Opdivo^®^) [98] and pembrolizumab (Keytruda^®^) [99], has given benefits to patients with early stage-II disease. Although antibodies block the downstream pathway of PD-1/PDL-1, they do not decrease the target protein’s transcription levels, which could result in a more efficient therapy [100]. Furthermore, the reactivation of T-cells and autoimmune inflammation caused by immune checkpoint inhibitors is responsible for skin rash, colitis, and inflammation of the pituitary glands [101]. Peptide nucleic acids (PNAs) can be a valid alternative to antibodies due to their unique antigene and antisense properties capable of inhibiting both PD-L1 transcription and translation [102]. PNAs are DNA/RNA synthetic analogs in which the phosphate–sugar backbone is replaced with a 2-([2-Aminoethyl]amino) acetic acid backbone, providing PNAs with higher stability to enzymatic degradation and a neutral spine. Unfortunately, PNAs show limited tissue accessibility, low water solubility, and an unfavorable pharmacokinetic profile, hindering their translation into clinics [103]. Terracciano et al. recently reported redox-responsive DNPs loaded with an anti-PD-L1 PNA for enhanced uptake in cancer cells and efficient protein downregulation [59] (Table 1). The PNA was bound to polyethylene glycol (PEG)-modified DNPs via a disulfide bond with a loading efficiency of 31% and released after bond reduction by glutathione in cancer cells. Due to the excess of glutathione in the tumor (2–20 mM) compared to normal tissues, the strategy shown by Terracciano et al. promoted PNA release in malignant cells with higher efficiency than in healthy samples, reducing off-target protein downregulation and toxicity. Moreover, DNPs improved the uptake of PNA in A549 cells already after 6 h of incubation (Figure 6a), yielding an internalization four-fold higher than the free PNA [59]. Managò et al. used Raman spectroscopy to determine the kinetics of internalization and localization of siRNA-loaded DNPs with an average diameter of 450 nm targeting cancer cells [104]. The Raman data confirmed that DNPs are internalized through endocytic uptake after 6 h of incubation with the cells (Figure 6b), followed by a complete internalization after 24 h (Figure 6b), leading to an efficient distribution of the nanocarrier in the cytoplasm within 72 h (Figure 6b) [104]. After reaching the cell cytoplasm, the oligonucleotide sequence released from the DNPs can exploit its function and downregulate the expression of target proteins with higher efficiency than the free SiRNA. The capability of DNPs to promote the internalization of low-water-soluble oligonucleotide-based drugs may be the key to their success in clinical trials. Interestingly, new findings on a rat model of colon carcinoma revealed the key role of B-cell lymphoma-2 (Bcl-2) overexpression in cancer escape from immune surveillance [105]. Thus, manipulating the expression of the Bcl-2 family members could also be involved in the sensitivity of CRC to the immune system eliminating cancer cells through the induction of cell death. Gene therapy using siRNA to downregulate the expression of Bcl-2 represents a promising tool in cancer treatment [106]. However, siRNAs are susceptible to enzymatic degradation and lack penetration capability due to their strong negative charge and high molecular weight. Martucci et al. modified DNPs with a mean diameter of 370 ± 20 nm with an Id-peptide recognized by the B-cell receptor (BCR) for the targeted delivery of the anti-Bcl-2 siRNA (DNPs-siRNA) into cancer cells [60] (Table 1). The delivered sequence from DNPs-siRNA decreased the expression levels of Bcl-2 mRNA by ~45% within 48 h of incubation, while the free siRNA downregulated the mRNA levels by only ~10%. The enhanced downregulation was provided by a higher internalization of the nano complex in non-permeabilized and permeabilized cells (Figure 6c,f) compared to the untreated cells (Figure 6d,f). The successful delivery of siRNAs from the DNPs and further cell internalization suggests a promising approach for RNA-interfering therapeutic applications to support immune system surveillance [60]. 

Unfortunately, few examples of DNPs targeting immunological checkpoints have been reported so far for treating CRC, likely due to the current understanding of their role in cancer evasion [107]. 

Zhao et al. developed cell membrane-camouflaged mesoporous silica NPs loaded with dacarbazine (DTIC) and modified with an anti-PD-1 antibody for immunotherapy in vivo [108]. The developed NPs suppressed cancer growth and prolonged the survival rate due to the synergistic tumor killing, activation of tumor-specific T cells, and regulation of the immunosuppressive tumor microenvironment. Reda et al. reported on polymer-coated mesoporous silica NPs delivering both volasertib and PD-L1 to synergize the effects of the drug and antibody, enhancing therapeutic efficacy and overcoming the dose-limiting toxicity of current PD-L1 inhibitors [109]. The developed delivery system led to cell cycle arrest and the generation of an anti-tumor immune response, both provided by a higher drug release in the targeted cells overexpressing PD-L1 than in healthy ones. 

Despite their potentiality as drug nanocarriers, the sol–gel production of mesoporous silica NPs requires high pressure, temperature, and prolonged reactions to remove the precursor’s tetraethyl orthosilicate (TEOS) or tetramethyl orthosilicate (TMOS) [110]. Nonetheless, TMOS is highly toxic by inhalation and very irritating to the skin and eyes, causing lesions in the nasal mucosa, kidney damage, and cytolysis in the red and white pulp of the spleen [111]. Due to these issues, replacing mesoporous silica NPs with natural diatom nanocarriers would represent an eco-friendly strategy to produce biocompatible nanocarriers targeting unbalanced immunological checkpoints in metastatic CRC. 

## 4. Conclusions

Cancer treatment has been revolutionized by the application of nanotechnology tools and techniques in the field of medicine, an approach called nanomedicine. The most-exploited substrates in nanomedicine are nanoparticles, representing a powerful approach for targeted drug delivery. Nanoparticles loaded with drugs can guarantee higher therapy specificity, lower doses, and fewer side effects compared to conventional treatments. Various nanoparticles have been tested for targeted drug delivery, including liposomes, micelles, and polymeric and porous nanoparticles. Among them, inorganic porous biosilica nanoparticles derived from diatoms have advantageous properties such as biocompatibility, high surface-to-volume ratio, and chemical stability under physiological conditions.

This review paper presented the latest results related to the use of diatomite nanoparticles for the treatment of colorectal cancer. The reported data clearly demonstrate the interesting potential of this robust technology in the field of cancer therapy, which makes it possible to overcome drawbacks of conventional medicine, especially in terms of drug distribution and toxicity. Diatomite nanoparticles have also been used as multifunctional nanodevices providing unpredictable information, such as quantification of the released drug in cells and correlation with the therapeutic effect in vitro.

Although many intriguing results were reported in this review, in vivo studies are still lacking. Currently, only a few studies have demonstrated the use of porous biosilica-based nanoparticles in small animal tumor models; among them, there are no studies on colorectal cancer. Unfortunately, the data available in the literature do not demonstrate the safety of diatomite nanoparticles for drug delivery. Diatom nanoparticles accumulate in the liver and kidneys due to non-biodegradability in body fluids, potentially increasing toxicity and limiting the dose of the nanocarrier that can be administered per day. Additional efforts are still needed to study the distribution of nanoparticles in the body and their biodegradability over time. The conversion of diatom biosilica into silicon replicas must be considered in advance when developing diatom-based nanotherapeutics because it determines the utility of these nanoparticles. Therefore, it is necessary to investigate the scale-up feasibility of nanocarriers with specific biomedical applications and reduced side effects. The translation of diatom biosilica into clinics will require the development of systematic in vivo studies using well-characterized and stable diatom nanotherapeutics in mice and animal models (e.g., zebra fish). Finally, the choice of the delivery routes (e.g., systemic, or local) and dosing strategy must be considered to decrease general or immune toxicity.
